# Collagen and Keratin Hydrolysates to Delay the Setting of Gypsum Plaster

**DOI:** 10.3390/ma15248817

**Published:** 2022-12-09

**Authors:** Constantin Voinitchi, Carmen Gaidau, Fanica Capatana Tudorie, Mihaela Niculescu, Maria Stanca, Cosmin-Andrei Alexe

**Affiliations:** 1 Department of Roads, Railways and Construction Materials, Technical University of Constructions Bucharest, Bulevardul Lacul Tei nr. 122, 020396 Bucharest, Romania; 2 Leather Research Department, Research and Development National Institute for Textiles and Leather-Division Leather and Footwear Research Institute, 93, Ion Minulescu Str., 031215 Bucharest, Romania

**Keywords:** leather waste, keratin waste, protein hydrolysates, by-products reclaiming, retardant admixture, gypsum plaster

## Abstract

Leather and wool waste represent a high concern due to the low level of valorization and circular economy demands for upcycling of biomass resources. Both biomasses can be easily processed as protein hydrolysates and used as functional additives due to the amphiphilic and tunable properties of collagen and keratin proteins. The chemical, physical, and structural investigations of collagen and keratin hydrolysate properties showed that the chelating abilities due to carboxylic groups can be exploited for gypsum retardant additives. The molecular weights and amino acid compositions of three different hydrolysates showed only slight influences on the setting time of gypsum; all three proteins delayed the setting time of gypsum between 60 and 120 min, as compared to the commercial plaster with a 30 min setting time. Higher molecular weight and more carboxylic active groups showed slight improvements in the setting time of mortars. The improved properties of keratin hydrolysate as compared to low molecular collagen hydrolysate were attributed to foaming and conductive properties. The mechanism of mortar setting delaying through calcium ions complexation by protein hydrolysates was shown by electric conductivity evolution of plasters with and without protein additives over time, supported by foaming properties, amino acid, and functional groups’ composition. Lower bending strength values for the higher concentration of proteins do not reduce the potential to use the protein hydrolysates as retardant additives in mortar fabrication.

## 1. Introduction

The leather industry represents the oldest ecological activity by processing the by-products of the food industry, animal hides, and skins. Globally processed hides and skins are estimated to be 7.2 million tons, saving the costs of dumping worth USD 950 million per year [1] and creating finished leathers with a value of about USD 32 billion [2].

The complex process of transforming organic and easily biodegradable materials into a very durable and fashionable product for different commodities generates important quantities of organic-based waste.

Thus, from a ton of raw bovine hides, only 26% represents the finished products [3], and the rest is waste with different compositions and structures: 22.0–27.5% rawhide trimmings, 16.0–33.6% fleshings, 20.4–26.3% tanned splits, 17.5–22.8% tanned trimmings, 0.5–0.7% leather buffing dust, and 5.8–6.8% finished leather trimmings [4,5].

When the mass balance in hide processing is made taking into consideration the collagen (the hide substance), the result shows that about 51% of the hide collagen input is waste, from which about 37% is tanned collagen and 13% is untanned hide substance [6].

In the past two decades, many signs of progress related to energy and water saving were recorded in leather processing, but the leather waste level has remained the same [7].

The stricter regulations related to organic waste landfill disposal [8] will increase the costs of waste management and will drive the research for development of new materials based on leather industry waste.

If untanned waste can be processed to manufacture collagen-based materials (gelatin, biomaterials, etc.), for tanned waste only leather board fabrication represents a viable route, most of the leather waste being disposed of in the landfill. The management of leather waste is presently considered one of the most difficult environmental problems [9]. The strategy of the chemical auxiliary companies is to design alternatives to chromium tanning materials by using chemicals conferring enough durability in wearing and improved biodegradability after the end-of-life cycle of leather products [10,11,12].

Wool is a by-product of sheep farms, and in recent years it has become waste due to its coarse quality and the unprofitability of its processing. A resource of 200,000 tons of wool is produced in Europe and only 25% is processed in the textile industry [13].

Keratin is known to be difficult to degrade, and the sulfur content makes it harmful to the environment to be burned [14]. Different projects demonstrated the potential of wool valorization as fertilizer after hydrolysis with superheated water [15] and alkaline-enzymatic hydrolyses [16,17] or in cosmetic and medical applications [18,19].

The addition of proteins in mortars has been known since ancient times to significantly improve the quality of mortars in terms of durability, plasticity, and strength. The emulsifier properties and foam formation in spherical bubbles contribute to the surface tension reduction of water and higher pressure resistance of mortars [20]. Hair (horse, yak, goat, bovine) is also still used in lime plaster and mortars to provide strength and crack resistance to historical buildings [21].

The use of leather waste in the construction materials industry was recently demonstrated by replacing sand in the composition of mortars with chromium-tanned waste (wet-blue shavings) encapsulated in polypropylene, up to 75% proportion [22]. The results showed a lower reduction in compression strength of mortar as compared to other experiments with polymer wastes (recycled plastic waste, polyethylene terephthalate, and polycarbonate) offering a solution for environmental concerns related to the leather industry.

A recent review on leather waste utilization as a polymeric material [23] presents research reports on asphalt improvement by adding 0.3% leather dust [24] in compositions with leather waste, or in Portland cement for paving blocks [25].

Kollamat is an extruded polymer with leather fiber waste content with heat and sound insulation characteristics and with the advantage of moisture regulation properties as compared to classical products based on petroleum-origin polymers [26].

Keratin hydrolysates prepared from horns and hooves in combination with carboxymethylcellulose have demonstrated a good anti-ice coating for cement-concrete road pavement [27], able to replace expensive materials.

The aim of the research was to demonstrate the potential of protein hydrolysates originating from leather industry waste and sheep breeding to be used as an additive for plaster setting retardation.

According to some references, substances such as sodium carbonate, sodium silicates, lime, glycerin, gelatin, bone glue, citric acid, sodium citrate, sugars, and polyvinyl alcohol are retardants for plaster setting [28,29,30,31]. In 2020 [31], 150,000,000 tons of gypsum were used worldwide for various purposes, including construction plasters, so that the plaster retardant additives can find a high potential for market exploitation.

The protein hydrolysates were prepared, characterized, and experimented for application as retarding additives for building construction plaster use. The optimum dosage and the effects on plaster strength were investigated along with the delaying mechanism by plaster suspension conductivity measurements and the evolution of temperature released by plaster hydrating. The hypothesis of calcium ions complexing by amphionic protein hydrolysates as a mechanism of delaying plaster setting time was shown by analyzing conductivity and complexation properties. Original findings are related to the correlation of different collagen and keratin hydrolysates, their properties, and the influence on the plaster setting time retarding process. The potential of upcycling a valuable protein waste generated by the leather industry and by animal breeders in agriculture was proved as an alternative circular technology in the context of very few publications on this subject.

## 2. Materials and Methods

### 2.1. Materials

The following materials were used in this study: residual tanned leather fragments were collected from SC Pielorex SA tannery (Jilava, Romania); wool was purchased from sheep farmers (Constanta, Romania); Borron SE (ethoxylated alkyl derivatives with 65% concentration) was supplied by SC Triderma SRL (Bucharest, Romania); calcium oxide hydrated (CaO* CaOH, MW = 81.371 g/mol) and sodium hydroxide (NaOH, MW 40.00 g/mol) was purchased from Cristal R Chim SRL (Bucharest, Romania); and construction gypsum with setting time of 7–15 min was purchased from Regips Sant-Gobain (Bucharest, Romania). Chemical reagents of analytical grade such as ammonia (25%), sodium carbonate, and tartaric acid were purchased from Chimreactiv SRL (Bucharest, Romania); ferrous chloride and ferrozine (3-(2-pyridyl)-5,6-bis(4-phenyl-sulfonic acid)-1,2,4-triazine) were purchased from Redox (Otopeni, Romania). Alcalase 2.4 L (protease from *Bacillus licheniformis* with 2.4 U/g) was purchased from Sigma-Aldrich (Bucharest, Romania).

### 2.2. Methods

#### 2.2.1. Protein Hydrolysates Preparation

**Figure 1:**
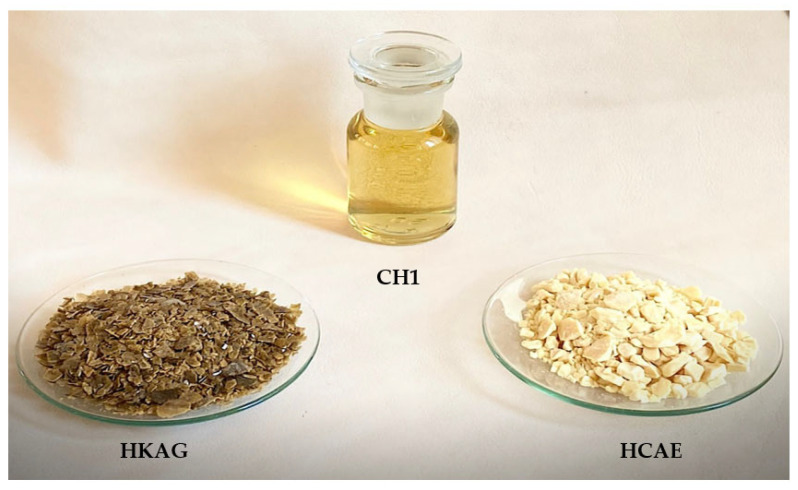
Protein additives prepared from leather and wool waste: CH1 (collagen-based hydrolysate, liquid), HCAE (collagen-based hydrolysate, solid), and HKAG (keratin-based hydrolysate, solid).

Three kinds of protein hydrolysates were prepared by alkaline and alkaline-enzymatic hydrolyses in order to be used as additives for plaster setting delayers. The three products are shown in Figure 1 and were prepared from leather waste (CH1 and HCAE) and from wool waste (HKAG). Their notation symbols followed their composition: H for hydrolysate, C for collagen, and K for keratin.

**Figure 2:**
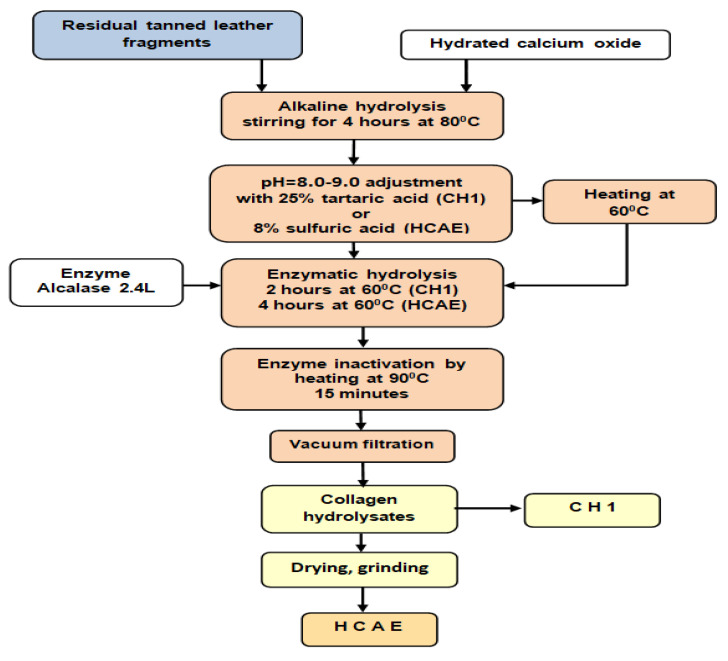
The flowchart of collagen hydrolysates preparation.

The collagen hydrolysates were prepared by compact alkaline-enzymatic hydrolysis according to Figure 2. The residual tanned bovine leather fragments were hydrolyzed in 600% water (w/v) with 10% CaO (w/w), for 4 h at a temperature of 80 °C, under mechanical stirring. The pH was adjusted to 8.0–9.0 value with 25% (w/w) tartaric acid solution for CH1 product and with 8% (v/w) sulfuric acid for HCAE; then, the reaction mass was heated at the temperature of 60 °C, and 1.0% Alcalase 2.4 L (w/w) was added under stirring regime for 2 h (CH1), and for 4 h (HCAE), respectively, at 60 °C. After enzymatic hydrolysis, the temperature was raised to 9 °C and it was maintained for 15 min for the enzyme deactivation. Subsequently, after filtration, the liquid collagen hydrolysate was collected (CH1). In addition, collagen hydrolysate liquid was dried by forced convection at a temperature of 65 °C, and after cooling, the dry matter was ground (HCAE).

**Figure 3:**
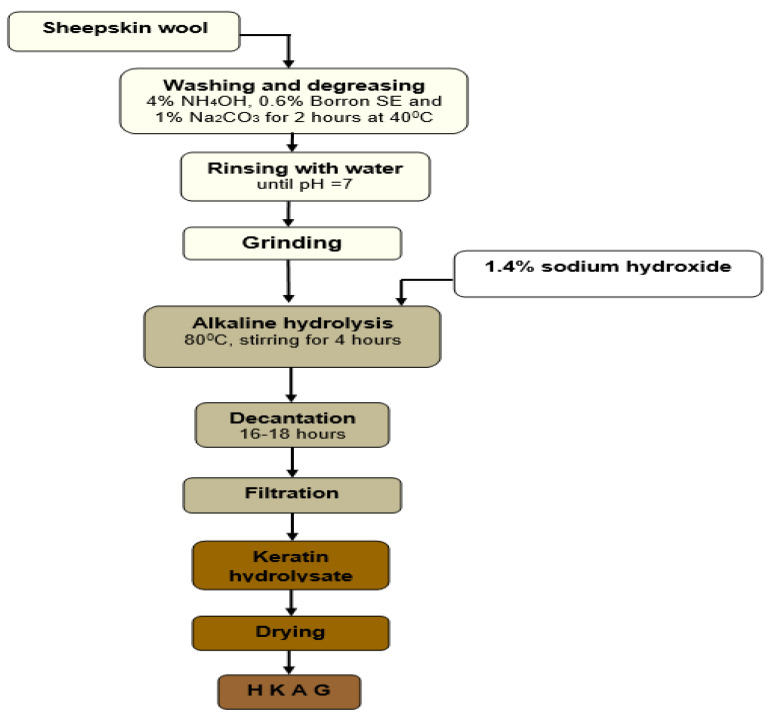
The flowchart of keratin hydrolysate preparation.

The keratin hydrolysate was prepared by alkaline hydrolysis according to Figure 3. The sheepskin residual wool was washed and degreased using 4% w/w NH_4_OH, 0.6% w/w Borron SE, and 1% w/w Na_2_CO_3_ for 2 h at 40 °C, followed by successive rinses with water until neutral pH was reached. The washed and degreased wool was chopped with a La Minerva bench grinder machine (Minerva Omega, Bologna, Italy). The cut wool was immersed in 900% w/w water at 80 °C, mixed with 1.4 % w/v sodium hydroxide solution in a stainless-steel vessel equipped with a mechanical stirrer and automatic temperature control (SC Caloris SA, Bucharest, Romania), for 4 h, when the wool was solubilized with 96% yield. After filtration, the keratin hydrolysate liquid was dried by forced convection at 65 °C when a product in the form of flakes was obtained (Figure 1).

#### 2.2.2. Collagen and Keratin Hydrolysates Characterization

The physical–chemical characteristics were analyzed according to standardized and in-house methods for volatile or dry matter (SR EN ISO 4684:2006), sulfated ash content (SR EN ISO 4047:2008), total nitrogen and protein content (SR ISO 5397:1996), pH (STAS 8619/3:1990), aminic nitrogen (ICPI method), cysteine and cystine sulfur (SR 13208:1994), chromium content (SR EN ISO 17294-2:2017 and SR EN ISO 16171:2017).

The results of the analyses were expressed as the average values of three determinations with standard deviation values.

The molecular weight and polydispersity of protein hydrolysates were determined by using an Agilent Technologies instrument (1260 model) (Agilent Technologies, Santa Clara, CA, USA) equipped with a PL aqua gel-OH MIXED-H column (7.5 × 300 mm, 8 µm) and multi-detection unit. The analysis conditions for gel permeation chromatography (GPC) were the flow rate of the mobile phase containing 1 mL min^−1^, injection volume of the sample 100 µL, and temperature of 35 °C for the detectors and column. Calculations of the Mw and number average molecular weight (Mn) were performed with the Agilent GPC/SEC Software (Version 1.1, Agilent Technologies, Santa Clara, CA, USA).

The electric conductivity of the three protein hydrolysates was determined for solutions with concentrations of 0.2% protein by using a conductometer (Consort C1020 multiparameter analyzer, Merelbeke, Belgium).

The metal ion chelating ability of protein hydrolysates was measured in order to explain the potential mechanism of delaying plaster setting induced by protein hydrolysates. The chelating method with FeCl_2_ was chosen as a model for the chelating ability of calcium ions due to the previous studies related to the behavior of ions with similar atom radii (Li^+^, Ni^2+^, Cu^2+^, Zn^2+^) in interaction with hydrated amino acids [32,33].

In this regard, the method [32,34] consisted of mixing 4.7 g protein hydrolysates (with a protein content of 0.23–0.29 mg/mL) with 0.1 mL of 2 mM FeCl_2_ and 0.2 mL of 5 mM ferrozine, followed by incubation for 20 min at room temperature. The ferrous ion chelating ability of protein hydrolysates was measured by UV-Vis absorbance (Jasco 550, ABL&E-JASCO Romania S.R.L., Cluj Napoca, Romania) at 562 nm in comparison with distilled water, and calculated according to the Equation (1):

(1) $$\mathrm{Fe}2^{+}\;\mathrm{chelating}\;\mathrm{ability}\;(\%)=\frac{A\;\mathrm{control}-A\;\mathrm{sample}}{A\;\mathrm{control}}\times 100$$

where A control and A sample are the absorbances of the control and sample at 562 nm.

The foaming properties of collagen and keratin hydrolysates were determined following the methods described by Sathe and Salunkhe (1981) [35]. The protein hydrolysate (0.1 g) was dissolved in 20 mL of distilled water. The pH of the solution was adjusted with 0.5 N NaOH or 0.5 N HCl. After reaching the optimum pH for the solutions (pH = 2; 4; 6; 8; 10), it was transferred into 100 mL Class A—measuring cylinder and was shaken manually with the same number of up and down movements for 30 s. Total volume was recorded immediately at t = 0 min, after 30 min and 60 min. Foam capacity and foam stability were calculated using the following Equations (2) and (3):

Foam capacity (%) = [(A − B)/B] × 100(2)

where A is the volume after whipping at t = 0 min (mL) and B is the volume before whipping (mL).

Foam Stability (%) = [(A − B)/B] × 100, where A is the volume at t = 30 min or 60 min (mL) and B is the volume before whipping (mL).

The results were reported as average values of three determinations.

The amino acid composition of protein hydrolysates was analyzed by HPLC chromatography using Amino Acid Analyser LC3000 (Sykam GmbH, Eresig, Germany), equipped with a polymeric cation exchanger column, post-column ninhydrin derivatization at 125 °C and photometric measurement at 570 nm. The results were monitored by Chromatography-Software ChromStar 6.0 (SCPA GmbH, Bremen, Germany) and were reported as mean of triplicate determinations.

The structural composition of protein hydrolysates was analyzed by ATR-FTIR spectroscopy using Bruker VERTEX 70 spectrometer (Ettlingen, Germany) with a 4 cm^−1^ resolution. The background and sample spectra were obtained in the 900–4000 cm^−1^ wavenumber range. Spectral processing was achieved with the OPUS and ORIGIN programs. For this investigation, CH1 product was dried in a Petri dish at 100 ± 2 in an oven (Memmert UF110, Essen, Germany) for 24 h, until a dried film was obtained.

#### 2.2.3. Gypsum Characteristics

Plaster preparation was prepared by determination of the water/plaster ratio for the paste of normal consistency according to the standard SR EN 13279-2: 2014 [36] by the sprinkling method. The paste of normal consistency was used to determine the setting time.

The start- and end-setting times were determined according to SR EN 13279-2:2014 by measuring the time from the preparation of the paste with standard consistency until it put up a certain resistance to the penetration of the Vicat needle, a period of time called setting time. It is expressed by the beginning of the setting, the end of the setting, and the set interval.

The plaster conductivity was determined on a suspension of plaster in water in a ratio of 1:5 or in diluted suspensions of the 3 protein hydrolysates with the same concentration as the normal consistency plasters, which means 0.1 g dry substance for 100 g plaster. The results were used to highlight the effect of the addition of delaying protein hydrolysates on the processes during the hydration of the plaster. The measurements were performed with a 3540 Bench Conductivity/pH Meter (Staffordshire, UK) and the results were the average of triplicate values.

The temperature variation was measured for the plaster paste of normal consistency made with distilled water or distilled water and delayed addition in a percentage of 0.1 g dry matter per 100 g of plaster. The results were used to highlight the effect of the addition of delaying protein hydrolysates on the processes during the hydration of the plaster.

The apparent density of the hardened mortar paste can be determined by the ratio between the mass of the material and the volume occupied by it. The mass was determined by weighing the sample brought to the constant mass, and the volume, by determining the dimensions of the parallelepiped that constituted the sample.

The influence of delaying additives on plaster density was assessed by preparing 10 sets of prismatic samples with dimensions of 160 × 40 × 40 mm that were left to harden in the laboratory atmosphere, after which they were dried in an oven at 40 °C until constant mass. The 10 sets were: 1 set of control samples from gypsum paste with water and 3 sets of prisms for the three protein hydrolysates with 3 dosages of 0.05%, 0.1%, and 0.2% relative to the mass of the plaster.

The bending resistance was analyzed according to SR EN 196-1 [37] by measuring the force that acts perpendicularly to the direction of the casting of the prism and will be applied continuously and evenly until the test piece breaks. The expression of results was given by the Equation (2):

P_f_ = 0.00234 × P, [N/mm^2^] (3)

where P is the breaking force at bending in N. The values were the average of triplicate measurements.

The compression strengths of new plasters were determined following the method from SR EN 196-1, immediately after bending resistance assessment on the halves of the plaster prisms. The result was the arithmetic mean of six determinations and was calculated according to the Equation (3):

R_c_ = F_c_/A, [N/mm^2^] (4)

where:

F_c_ is the maximum breaking load force in N;

A is the area of the test surface in mm^2^.

## 3. Results

### 3.1. Protein Hydrolysates Characteristics

In Table 1, the main physical–chemical characteristics are presented for the three protein hydrolysates (Figure 1). The three kinds of proteins showed different characteristics according to the raw materials and preparation methods. The protein content of samples was above 74% and the molecular weights were from 2530 Da to 25,069 Da, in agreement with aminic nitrogen content, which showed higher values for lower molecular weight, due to the collagen molecule breaking under alkaline and enzymatic hydrolyses, which was more intense for the HCAE product. The content in cysteine and cystine sulfur of keratin hydrolysate showed that even though the wool was completely solubilized, the hydrolyses did not destroy the specific amino acids. Electric conductivity values of protein hydrolysates were found to be in correlation with ash content.

**Figure 4:**
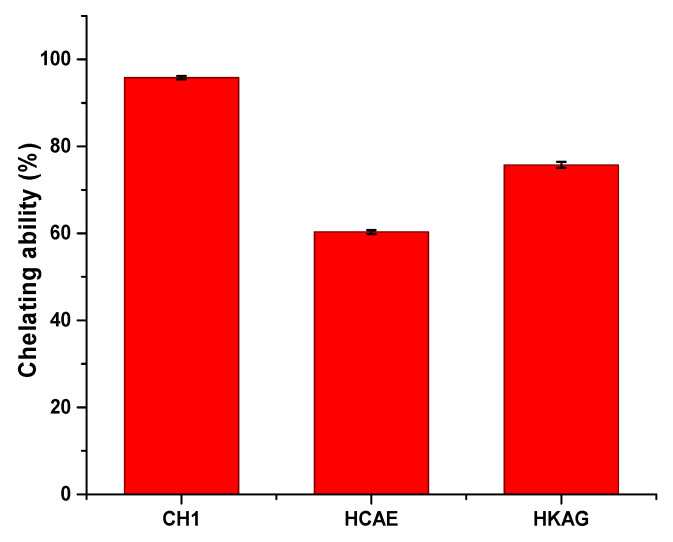
The chelating ability of protein hydrolysates: CH1, HCAE, and HKAG.

The chelating ability of the three protein hydrolysates is presented in Figure 4 and shows values that can be classified in the following order: CH1 > HKAG > HCAE.

**Figure 5:**
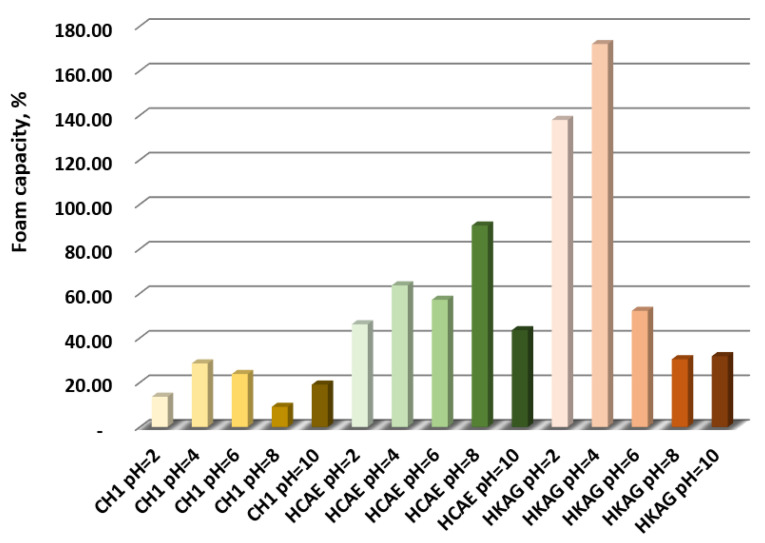
The foaming capacity of the protein hydrolysates.

The foam capacity is presented in Figure 5 and shows a high value for keratin hydrolysate (HKAG), followed by collagen hydrolysates, HCAE and CH1. The influence of pH value on foaming capacity was obvious and different for every protein hydrolysate.

The foam stability presented in Table 2 also shows a dependency on pH value and a more stable keratin hydrolysate foam as compared to collagen hydrolysates foams.

**Figure 6:**
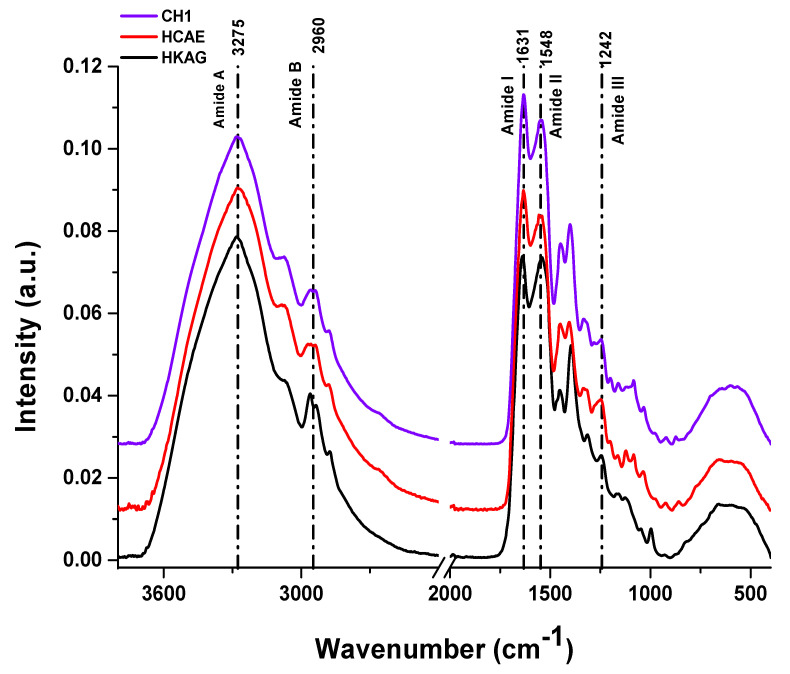
ATR-FTIR spectra of CH1, HCAE, and HKAG protein hydrolysates.

The ATR-FTIR spectroscopy analysis results are presented in Figure 6.

The ATR FT-IR spectra of protein hydrolysates showed the presence of specific major amide bands providing information on secondary structure that was still preserved after the extraction processes. The band Amide A was associated with the N-H group stretching vibrations and occurred in the range of 3300 cm^−1^ wavenumbers. The shift to lower frequencies (3275 cm^−1^) was due to involvement of NH groups of peptides in the formation of hydrogen bonds [38]. The Amide B band in the range 2924–2928 cm^−1^ was associated with the asymmetric stretching vibration of =CH and –NH_3_^+^, and the shift at 2960 cm^−1^ in protein hydrolysates was attributed to the increase of free NH-NH_3_^+^ clusters in the N-terminal lysine residues [39].

The Amide I peak was associated with the stretching vibration of C=O (1600–1700 cm^−1^) involved in hydrogen bonds connecting collagen chains. The shift of the Amide I band of collagen hydrolysates from 1631 cm^−1^ to 1640 cm^−1^ for keratin hydrolysate can be associated with more ordered keratin molecules due to the extraction process, without enzyme, and probably stronger intermolecular bonds [40]. The Amide II band, which can be found in 1510 cm^−1^ and 1580 cm^−1^ regions, was associated with the N-H in-plane bending and C-N stretching vibration. The Amide II peaks of collagen hydrolysates at 1546 cm^−1^ were found to be at higher wavenumbers than those for HKAG (1537 cm^−1^), due to stronger hydrogen bonds in keratin hydrolysate [41]. The Amide III peak was complex and related to the intermolecular interactions involving C-N stretching and N-H in-plane bending vibration due to amide links and C-C stretching and CH bending vibrations [42], as well as absorptions from wagging vibrations from CH_2_ groups from glycine backbone and proline side-chains. The higher intensity of the Amide III peak of HCAE and CH1 as compared to HKAG can be correlated with more bonds between glycine backbone and proline side-chains [43], in correlation with higher content of glycine and proline of collagen hydrolysates (Table 3). The ratios of Amide I/ Amide II of 1 [44,45] and Amide III/1440 [41] of 0.8 showed that the protein hydrolysates still have ordered moieties with preserved helix secondary structure after extraction processes.

The amino acid composition of the collagen and keratin hydrolysates is presented in Table 3 (and Tables S1–S3) and showed that the most abundant amino acids were glycine, proline, glutamic acid, hydroxyproline, alanine, and arginine for collagen hydrolysates, and glutamic acid, leucine, asparagine, glycine, proline, arginine, and valine for keratin. The cysteine acid was specific for keratin and confirmed the chemical analysis that showed that even though the solubilization was complete, the cysteine and cystine sulfur were still present. It is worth mentioning that 20 amino acids were identified in collagen hydrolysates and 18 (ornithine is a derivative of arginine) were identified in keratin hydrolysate. The amino acid profiles of collagen hydrolysates were very similar; more hydroxyproline can be seen in the case of the CH1 product. Meanwhile, higher concentrations of glutamic acid leucine and valine were found in keratin hydrolysate.

At the alkaline and neutral pH, amino acids (aspartic and glutamic acids [46]) were negatively charged and bound calcium positive charged ions, according to the reaction (5), thus retarding the hydration of CaSO_4_.



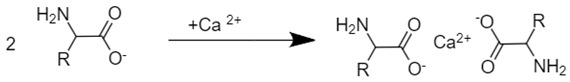

(5)


### 3.2. Delaying Setting Time of Plaster with Protein Hydrolysates Additives Concentrations

**Figure 7:**
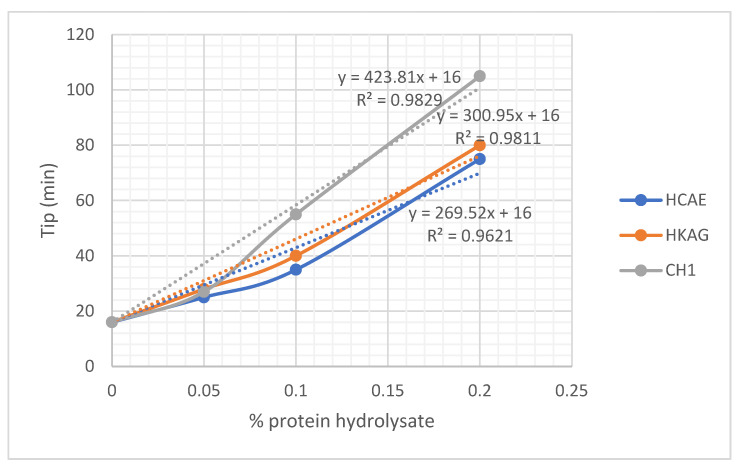
Variation of initial time for plaster setting as a function of protein hydrolysate concentration. Dotted lines represent a linear estimation of initial setting time as a function of hydrolysates dosage w/w. For the case when no hydrolysates were used, initial and final setting time is for gypsum plaster alone (16 min).

**Figure 8:**
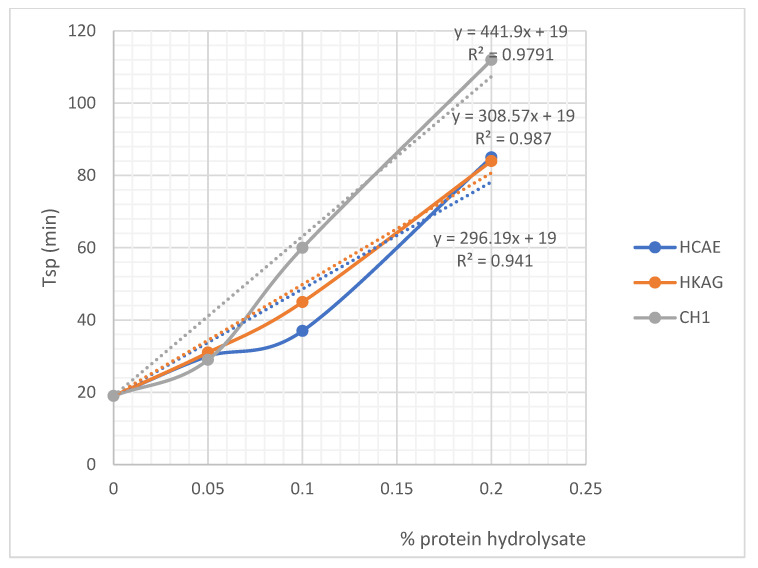
Variation of final time for plaster setting as a function of protein hydrolysate concentration. Dotted lines represent a linear estimation of final setting time as a function of hydrolysates dosage w/w. For the case when no hydrolysates were used, initial and final setting time is for gypsum plaster alone (19 min).

Figure 7 and Figure 8 and Table S4 show the variation of the initial (Tip) and final (Tsp) time of plaster setting as a function of protein hydrolysate concentration. The setting time of the gypsum mixed with 0.20 w/w % protein hydrolysates, HCAE and HKAG, showed almost similar values, with slightly higher values for keratin hydrolysate additive. The setting time was higher for the collagen hydrolysate CH1 in a liquid state, which can be an economical advantage due to the lower energy consumption. The results of the experiments confirmed the potential of protein hydrolysates to be used as retarders in plaster setting.

### 3.3. The Conductivity of Gypsum Suspensions in Water

**Figure 9:**
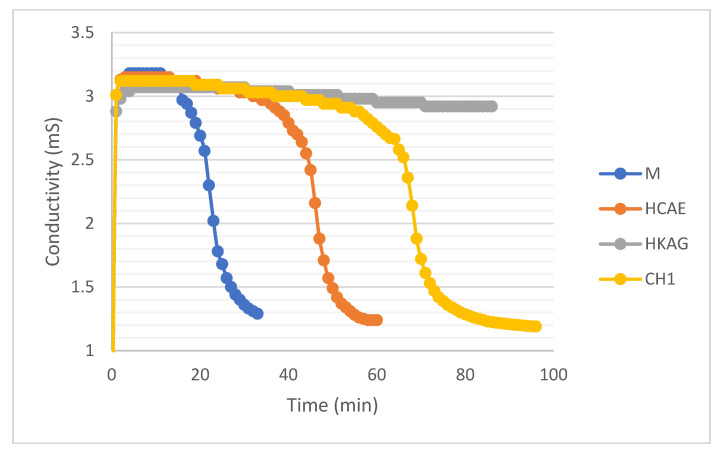
Conductivity evolution over time for the four plaster solutions (M-control sample).

Compositions made of 4 g plaster in 100 mL liquid-distilled water or water and solutions of the three protein hydrolysates were tested, so that the concentration of the dry matter was the same: 0.016 g/100 mL liquid. The results are shown in Figure 9.

### 3.4. The Evolution of Temperature in Gypsum Paste

**Figure 10:**
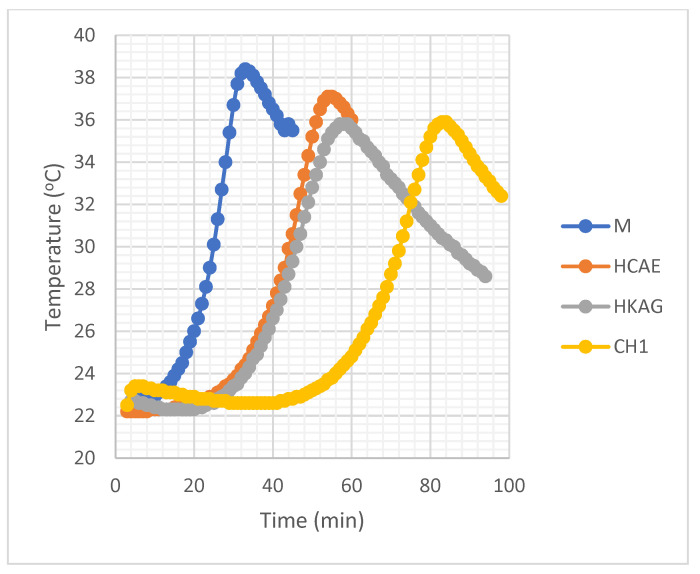
The evolution of the temperature in the gypsum paste in the quasi-diabatic precinct (M-Control sample).

The variation of the temperature of the gypsum pastes without addition and with addition of 0.1% protein hydrolysates is presented in Figure 10.

### 3.5. The Hardened Paste Density

Ten sets of prismatic samples with dimensions of 160 × 40 × 40 mm were prepared and left to harden in the atmosphere of the laboratory, after which they were dried in an oven at 40 °C until constant mass. The 10 sets were: 1 set of control samples of gypsum paste with water and 3 sets of prisms for the 3 wastes treated with 3 dosages each: 0.05%, 0.1%, and 0.2% of the mass of the plaster. The results of the bulk density determinations are presented in Table 4.

### 3.6. Bending and Compressive Strengths of Plasters

**Figure 11:**
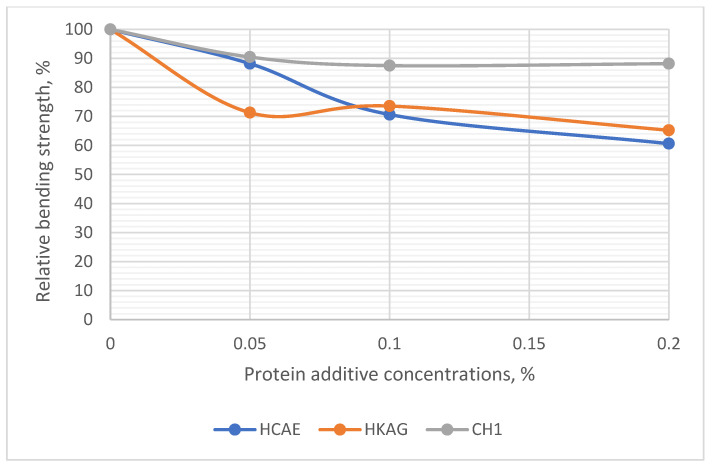
Relative bending strength of plasters with different concentrations of protein additives.

**Figure 12:**
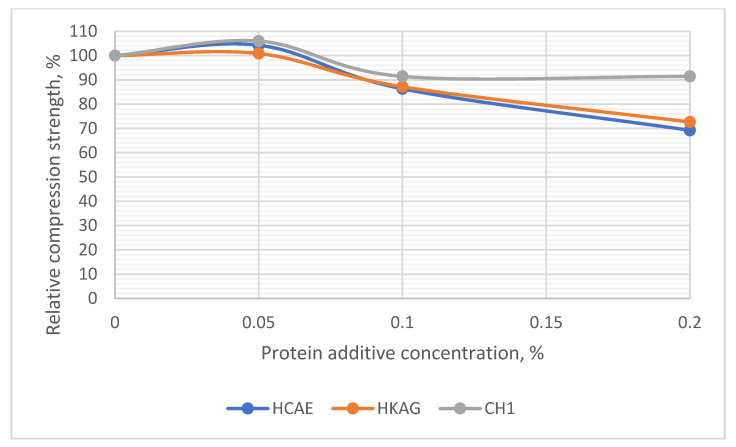
The relative compression strength of plasters with different concentrations of protein additives.

The results of determining the bending and compressive strengths after 7 days of hardening and drying are presented in Figure 11 and Figure 12 and Table S5.

## 4. Discussion

There were significant increases in the start time of setting, even at very low concentrations of protein hydrolysates (0.05%); acceptable setting time values were reached for concentrations of 0.10% and 0.20%, respectively, when it reached values of about one hour, enough time to implement even larger amounts of paste. It was observed that all three protein hydrolysates had similar properties; the delaying action on the setting time seemed to increase approximately linearly with the dosage, and it was possible to establish relationships between the setting time and dosage with a correlation coefficient of over 0.96. The strongest delaying action seemed to have the liquid dispersion of CH1 collagen, followed by relatively close values for the HKAG keratin hydrolysate and the HCAE solid collagen hydrolysate, in this order. The behavior of protein hydrolysates as plaster setting r retardants can be attributed to the amphiphilic and chelating ability of protein hydrolysates that is dependent on hydrolyses conditions and molecular weights [32,47]. The values of the chelating ability of 95.80%, 75.74%, and 60.30%, determined for the three protein hydrolysates, were in correlation with their delaying setting time performances: CH1 > HKAG > HCAE. The complexing of Ca^2+^ by carboxylic groups of the protein hydrolysates, also presented by other authors [27] as the basic mechanism of plaster setting delaying time, was confirmed in our research by complexing ability determinations in correlation to amino acid composition and ATR-FTIR spectra analysis. The complexing ability values found for collagen and keratin hydrolysates (75.74–95.80%) were in line with similar values (75%) reported for other protein hydrolysates extracted with proteinase enzymes [34]. The retardation performances followed the order CH1 > HKAG > HCAE. The protein hydrolysates, CH1, with the highest molecular weight of 25,069 Da showed a higher performance in delaying plaster setting time, followed by keratin hydrolysate with 13,433 Da and very close to HCAE with the lowest molecular weight of 2530 Da. The results were in agreement with other authors’ findings related to the collagen hydrolysate with a molecular weight of 10,000–30,000 Da with the best performances on plaster setting time delaying [27]. Other literature data showed that the collagen mineralization through Ca^2+^ chelating occurs not only to ionized carboxylic side chains but also to the carbonyl peptide chain [45], which can explain the performance of higher molecular weight collagen hydrolysate, CH1, in delaying plaster setting time as compared to HCAE, with a lower molecular weight and lower hydroxyproline concentration.

The results suggest that the easy processing technology for protein hydrolysate (Figure 2 and Figure 3) can lead to performance of plaster setting time, able to save costs for atomization [27] or oven drying of the final product.

In principle, the conductivity of the solution should increase from values close to 0 to a maximum value due to the passage in the solution Ca^2+^ and SO_4_^2−^ ions, after which, when the solubility limit is reached, there will be a significant decrease, due to ion losses embedded in the crystal lattice. This development was very clear for the suspension of plaster in water in Figure 9. It was observed that the maximum conductivity recorded for the suspensions with protein hydrolysates was slightly lower, which shows that there may be an interaction with one of the two ionic species, Ca^2+^ and SO_4_^2−^, in the solution, probably Ca^2 +^ due to the protonated amino acids at alkaline pH values and chelating ability (Figure 4). There was also a significant delay in the onset of dihydrate crystallization, marked by a sharp decrease in the conductivity of the suspension, which seemed to be the plausible mechanism for increasing the onset time. The most effective retarder appeared to be HKAG, followed by CH1 and HCAE, as seen in Figure 9 for low protein hydrolysate concentrations—below 0.05% of the mass of the plaster.

There were two maximums of heat release: a smaller one specific for the passage of Ca^2+^ and SO_4_^2−^ ions in solution accompanied by their hydrating heat, and a higher one due to the release of a part of the network energy of the newly formed dihydrate. Figure 10 shows that the maximum heat release of the dihydrate network decreased in intensity and was shifted to higher values of time, which confirmed the delaying effect of the addition of protein hydrolysates.

The setting time delay was reported in the case of using nano-fibrillated bacterial cellulose originating from fermented sugar beet and sugar cane and was attributed to the sugar influence by inhibition of hydration reaction [48]. The most probable mechanism of the plaster setting time delay was the hindering of the calcium sulfate hemihydrate formation in the initial setting stage and dihydrate form in the final stage setting by calcium complexing through protein carboxylic groups, in an ionized state, at higher pH values (9.25–10.30, Table 1) as compared to the isoelectric point pH (around 4 value) and the lower access to the water molecules bound to the hydrophilic peptides.

The apparent dry densities after 7 days had practically the same value as the control; only the sample containing HKAG seemed to have a slightly lower density, possibly due to an air entrainment effect that may offer a new application, after a thorough study. The higher foaming capacity of HKAG as compared to the collagen hydrolysates confirmed this hypothesis (Figure 5) for the initial moment, the foam stability after 30 min being similar for all protein hydrolysates (Table 2).

There were decreases in bending strength between 10 and 40% compared to the gypsum control paste at a dosage of 0.2 w/w % of protein hydrolysate. Although they were substantial, they do not pose a threat, as the plastering works are not structural.

The decrease in resistance associated with delay and hardening can be seen in Table S5, where decreases were observed for higher dosages, slightly increasing with its value. At low dosages, the compressive strength did not seem to be affected, but rather had slightly higher values than the control (Figure 12).

It was shown by direct and indirect experiments that all three wastes have the ability to delay setting and bring setting time to reasonable values of around one hour upon addition of 0.1–0.2 w/w % of the mass of the plaster as dry matter. Through the direct determinations of the start and end time of the setting, a delay of 1–1.5 h was found upon addition of 0.2 w/w % for all three protein hydrolysates. The determinations of the variation of the paste temperature and of the conductivity of the gypsum suspensions in the water showed a slight decrease in the intensity of the phenomena accompanied by a time lag of them, in accordance with the delay of the setting found directly. The additives probably act by delaying the crystallization of calcium sulfate dihydrate by chelating calcium ions, as shown by chelating ability analyses and the adsorption on the crystallization germs formed as presented by other authors [27]. The associated decreases in mechanical strength are of the order of 10–40% of the value of the control paste and are not significant due to the non-structural uses of the plaster.

Future research will be devoted to manufacturing other construction materials with collagen and keratin hydrolysates extracted from waste biomasses.

## 5. Conclusions

Waste biomasses originating from the leather industry and sheep breeders, collagen-based leather waste, and coarse wool, respectively, were processed following unsophisticated methods in view of recovering them as gypsum plaster retardant additives, a highly demanded material worldwide. The characterization of collagen and keratin hydrolysates and gypsum plasters prepared with these protein additives showed that the retarding time of 1–1.5 h can be reached with acceptable influences on gypsum plaster properties. The mechanism of delaying the time of plaster adhesion was attributed to the chelating ability of protein hydrolysates of calcium ions, responsible for calcium sulfate hydration. The recovery and recirculation of collagen-based waste were demonstrated for the benefit of both the leather and construction industries. Keratin hydrolysate processed from coarse wool can also find successful applications in construction materials fabrication.

## Figures and Tables

**Table 1:** Physical–chemical characteristics of protein hydrolysates.

Characteristics	Collagen Hydrolysate Dispersion, CH1	Collagen Hydrolysate Granules, HCAE	Keratin Hydrolysate, Flakes, HKAG
Dry matter, %	7.09 ± 0.42	-	-
Volatile substances, %	-	8.28 ± 0.42	10.97 ± 0.42
Total nitrogen, %	15.55 ± 0.66	13.31 ± 0.66	12.50 ± 0.66
Protein, %	87.45 ± 2.26	74.79 ± 2.26	75.75 ± 2.26
Aminic nitrogen, %	0.98 ± 0.12	1.40 ± 0.12	0.75 ± 0.12
Average molecular weight, Da	25,069	2530	13,433
Polydispersity	1.01	1.00	1.39
Total sulfated ash, %	11.98 ± 0.27	7.25 ± 0.27	14.40 ± 0.27
Cr, mg/L	39.30 ± 4.11		-
Cr, mg/kg		78.68 ± 9.20	-
pH (1:10)	10.10 ± 0.10	9.25 ± 0.10	10.30 ± 0.10
Cysteine, %	-	-	1.45 ± 0.03
Cystine sulfur, %	-	-	0.39 ± 0.03
Electric conductivity, mS/cm	5.96 ± 0.15	3.96 ± 0.26	7.12 ± 0.20

**Table 2:** The foam stability of protein hydrolysates, CH1, HCAE, and HKAG.

Sample	Foam Stability, %		
	30 min	60 min	90 min
CH1 pH = 2	0	0	0
CH1 pH = 4	0	0	0
CH1 pH = 6	4.76	0	0
CH1 pH = 8	0	0	0
CH1 pH = 10	0	0	0
HCAE pH = 2	0	0	0
HCAE pH = 4	0	0	0
HCAE pH = 6	0	0	0
HCAE pH = 10	0	0	0
HKAG pH = 2	6.90	3.45	3.45
HKAG pH = 4	4	4	0
HKAG pH = 6	0	0	0
HKAG pH = 8	0	0	0
HKAG pH = 10	0	0	0

**Table 3:** Amino acid composition of protein hydrolysates.

Amino Acid, g/100 g Protein	CH1	HCAE	HKAG
Asp (D/N)	5.60 ± 0.04	5.38 ± 0.09	8.30 ± 0.12
Hyp	10.69 ± 0.10	9.36 ± 0.08	-
Thr (T)	0.82 ± 0.007	0.84 ± 0.008	3.11 ± 0.01
Ser (S)	1.41 ± 0.002	1.37 ± 0.002	3.59 ± 0.009
Glu (E/Q)	10.72 ± 0.09	10.41± 0.09	19.21 ± 0.09
Pro (P)	14.09 ± 0.13	14.77 ± 0.12	7.49 ± 0.20
Gly (G)	23.28 ± 0.23	25.08 ± 0.23	7.66 ± 0.25
Ala (A)	9.86 ± 0.09	10.29 ± 0.08	6.54 ± 0.10
Val (V)	2.42 ± 0.02	2.36 ± 0.02	7.14 ± 0.01
Met (M)	0.99 ± 0.005	0.98 ± 0.005	1.23 ± 0.004
Ile (+allo-Ile) (I)	1.42 ± 0.005	1.44 ± 0.005	4.30 ± 0.002
Leu (L)	2.89 ± 0.015	2.80 ± 0.015	9.74 ± 0.005
Tyr (Y)	0.23 ± 0.009	0.29 ± 0.008	1.94 ± 0.003
Phe (F)	2.00 ± 0.002	1.89 ± 0.002	3.97± 0.001
His (H)	0.78 ± 0.006	0.66 ± 0.007	1.32 ± 0.003
Hyl	1.00 ± 0.001	0.94 ± 0.001	-
Ornithine	1.26 ± 0.002	1.05 ± 0.002	2.04 ± 0.001
Lys (K)	3.08 ± 0.003	3.10 ± 0.003	2.45 ± 0.004
NH3	0.58 ± 0.002	0.59 ± 0.002	1.42 ± 0.001
Arg (R)	6.88 ± 0.006	6.40 ± 0.007	7.33 ± 0.004
Cys(O3H)	-	-	0.88 ± 0.001
Cys	-	-	0.34 ± 0.001

**Table 4:** Apparent density for gypsum paste with various protein additives.

Additive Concentration, %	Apparent Density, kg/m^3^		
	HCAE	HKAG	CH1
0.00	1168	1168	1168
0.05	1180	1153	1168
0.10	1163	1154	1171
0.20	1158	1163	1169

## Data Availability

The data presented in this study are available on request from the corresponding author.

## Supplementary Materials

The following supporting information can be downloaded at: https://www.mdpi.com/article/10.3390/ma15248817/s1, Table S1: Amino acid composition of collagen hydrolysate CH1; Table S2: Amino acid composition of collagen hydrolysate HCAE; Table S3: Amino acid composition of collagen hydrolysate HKAG; Table S4: Time delay of plaster setting for different concentrations of protein additives, at the initial setting time (Tip) and at the final setting time (Tsp); Table S5: Bending and compressive strength of plasters with different concentrations of protein additive.
