# Supplementary material for: Collagen and Keratin Hydrolysates to Delay the Setting of Gypsum Plaster
Source: Materials (Basel). 2022 Dec 9;15(24):8817. doi: 10.3390/ma15248817 (PMC9785065; doi:10.3390/ma15248817)
Supplement: Supplementary file 1 [file materials-15-08817-s001.zip › materials-2037901-supplementary.pdf]

materials

Article

Collagen and Keratin Hydrolyzates to Delay the Adhesion of Mortars

Constantin Voinitchi[1], Carmen Gaidau[2,*], Fanica Capatana Tudorie[1], Mihaela Niculescu[2], Maria Stanca[2], and Cosmin-Andrei Alexe [2]

1 Department of Roads, Railways and Construction Materials, Technical University of Constructions Bucharest, Bulevardul Lacul Tei nr. 122, Bucharest, 020396, Romania
2 Leather Research Department, Research and Development National Institute for Textiles and Leather-Division Leather and Footwear Research Institute, 93, Ion Minulescu Str., 031215, Bucharest, Romania
* Correspondence: carmen.gaidau@icpi.ro

Table S1. Amino acid composition of collagen hydrolysate CH1

Amino acid	Measure value in nmol	Calc. value in µg/g
Asp (D/N)	24,111.16 ± 0.04	3.21
Hyp	46,690.52 ± 0.10	6.12
Thr (T)	3,949.74 ± 0.007	0.47
Ser (S)	7,691.59 ± 0.002	0.81
Glu (E/Q)	41,734.00 ± 0.09	6.14
Pro (P)	70,100.48 ± 0.13	8.07
Gly (G)	177,572.31 ± 0.23	13.33
Ala (A)	63,357.56 ± 0.09	5.65
Val (V)	11,852.07 ± 0.02	1.39
Met (M)	3,808.54 ± 0.005	0.57
Ile (+ allo-Ile) (I)	6,214.03 ± 0.005	0.82
Leu (L)	12,615.93 ± 0.015	1.65
Tyr (Y)	721.16 ± 0.009	0.13
Phe (F)	6,943.88 ± 0.002	1.15
His (H)	2,881.01 ± 0.006	0.45
Hyl	3,516.57 ± 0.001	0.57
Ornithin	5,441.47 ± 0.002	0.72
Lys (K)	12,047.07 ± 0.003	1.76
NH3	19,472.04± 0.002	0.33
Arg (R)	22,613.83 ± 0.006	3.94
TOTAL	57,268.23	100

Table S2. Amino acid composition of collagen hydrolysate HCAE

Amino acid	Measure value in nmol	Calc. value in μg/g
Asp (D/N)	9,610 ± 0.09	40,663.13
Hyp	16,974 ± 0.08	70,758.16
Thr (T)	1,671 ± 0.008	6,327.20
Ser (S)	3103.51 ± 0.002	10,368.38
Glu (E/Q)	16823.75 ± 0.09	78,690.18
Pro (P)	30,521 ± 0.12	111,707.71
Gly (G)	79,480 ± 0.23	189,678.55
Ala (A)	27,477 ± 0.08	77,829.07
Val (V)	4788.38 ± 0.02	17,833.12
Met (M)	1,560 ± 0.005	7,400.23
Ile (+ allo-Ile) (I)	2,616 ± 0.005	10,909.73
Leu (L)	5075.16 ± 0.015	21,163.17
Tyr (Y)	381 ± 0.008	2,192.90
Phe (F)	2,721 ± 0.002	14,289.61
His (H)	1,011 ± 0.007	4,987.09
Hyl	1,382 ± 0.001	7,124.37
Ornithin	1,892 ± 0.002	7,950.76
Lys (K)	5,048 ± 0.003	23,459.62
NH3	8,256 ± 0.002	4,469.47
Arg (R)	8,749 ± 0.007	48,427.55
TOTAL	756,230.02	100

Table S3. Amino acid composition of collagen hydrolysate HKAG

Amino acid	Measure value in nmol	Calc. value in µg/g	Weight in %
Cys(O3H)	3,135.40 ± 0.001	5,453.57	0.88
Asp (D/N)	26,977.13 ± 0.12	51,546.93	8.30
Thr (T)	11,284.73 ± 0.01	19,297.67	3.11
Ser (S)	14,787.65 ± 0.009	22,309.49	3.59
Glu (E/Q)	56,477.36 ± 0.09	119,290.15	19.21
Pro (P)	28,121.27 ± 0.20	46,478.54	7.49
Gly (G)	44,117.49 ± 0.25	47,545.15	7.66
Ala (A)	31,722.49 ± 0.10	40,576.45	6.54
Val (V)	26,355.12 ± 0.01	44,323.72	7.14
Cys	1,216.86 ± 0.001	2,116.55	0.34
Met (M)	3,563.47 ± 0.004	7,633.07	1.23
Ile (+ allo-Ile) (I)	14,164.76 ± 0.002	26,673.04	4.30
Leu (L)	32,100.07 ± 0.005	60,446.27	9.74
Tyr (Y)	4,631.71± 0.003	12,047.72	1.94
Phe (F)	10,403.83 ± 0.001	24,672.10	3.97
His (H)	3,692.41 ± 0.003	8,224.68	1.32
Ornithine	6,684.29 ± 0.001	12,681.90	2.04
Lys (K)	7,247.45 ± 0.004	15,210.10	2.45
NH3	36,103.84 ± 0.001	8,826.67	1.42
Arg (R)	18,219.65 ± 0.004	45,542.59	7.33
TOTAL	381,006.98	664,308.89	100.00

Table S4. Time delay of plaster setting for different concentrations of protein additives, at the initial setting time (Tip) and the at the final setting time (Tsp).

Additive concentration, %	Setting time	CH1, min	HCAE, min	HKAG, min
0.00	T_{ip}	16	16	16
	T_{sp}	19	19	19
0.05	T_{ip}	27	25	28
	T_{sp}	29	30	31
0.10	T_{ip}	55	35	40
	T_{sp}	60	37	45
0.20	T_{ip}	105	75	80
	T_{sp}	112	85	84

Table S5. Bending and compressive strength of plasters with different concentrations of protein additives

Additive concentration, %	Bending strength, N/mm^2			Compressive strength, N/mm^2		
	HCAE	HKAG	CH1	HCAE	HKAG	CH1
0.00	6.10	6.10	6.10	11.7	11.7	11.7
0.05	5.38	4.35	5.52	12.2	11.8	12.4
0.10	4.31	4.49	5.34	10.1	10.2	10.7
0.20	3.70	3.98	5.38	8.1	8.5	10.7
